# Carnitines as Mitochondrial Modulators of Oocyte and Embryo Bioenergetics

**DOI:** 10.3390/antiox11040745

**Published:** 2022-04-08

**Authors:** Martina Placidi, Giovanna Di Emidio, Ashraf Virmani, Angela D’Alfonso, Paolo Giovanni Artini, Anna Maria D’Alessandro, Carla Tatone

**Affiliations:** 1 Department of Life, Health and Environmental Sciences, University of L’Aquila, 67100 L’Aquila, Italy; martina.placidi@graduate.univaq.it (M.P.); giovanna.diemidio@univaq.it (G.D.E.); angela.dalfonso@univaq.it (A.D.); carla.tatone@univaq.it (C.T.); 2 Alfasigma B.V., 3528 BG Utrecht, The Netherlands; ashraf.virmani@alfasigma.com; 3 Division of Gynecology and Obstetrics, Department of Clinical and Experimental Medicine, University of Pisa, 56126 Pisa, Italy; pg.artini@gmail.com

**Keywords:** l-carnitine (LC), propionyl-l-carnitine (PLC), acetyl-l-carnitine (ALC), mitochondria, embryo, oocyte, beta-oxidation, bioenergetics

## Abstract

Recently, the importance of bioenergetics in the reproductive process has emerged. For its energetic demand, the oocyte relies on numerous mitochondria, whose activity increases during embryo development under a fine regulation to limit ROS production. Healthy oocyte mitochondria require a balance of pyruvate and fatty acid oxidation. Transport of activated fatty acids into mitochondria requires carnitine. In this regard, the interest in the role of carnitines as mitochondrial modulators in oocyte and embryos is increasing. Carnitine pool includes the un-esterified l-carnitine (LC) and carnitine esters, such as acetyl-l-carnitine (ALC) and propionyl-l-carnitine (PLC). In this review, carnitine medium supplementation for counteracting energetic and redox unbalance during in vitro culture and cryopreservation is reported. Although most studies have focused on LC, there is new evidence that the addition of ALC and/or PLC may boost LC effects. Pathways activated by carnitines include antiapoptotic, antiglycative, antioxidant, and antiinflammatory signaling. Nevertheless, the potential of carnitine to improve energetic metabolism and oocyte and embryo competence remains poorly investigated. The importance of carnitine as a mitochondrial modulator may suggest that this molecule may exert a beneficial role in ovarian disfunctions associated with metabolic and mitochondrial alterations, including PCOS and reproductive aging.

## 1. Introduction

Recently, growing awareness of the role of lifestyles, physical exercise, and aging as fertility determinants has driven research interest towards the importance of energy metabolism in the reproductive process [1,2]. Mammalian oocytes initiate meiosis in the embryonic ovary, arrest meiotically for decades, and resume meiosis at time of ovulation after an extensive growth phase. Thus, maintenance and regulation of energetic homeostasis is of particular importance for the biological competence of the mature oocyte. To this end, the oocyte relies on numerous mitochondria, which host most of metabolic processes required for its energetic demand. It has also been shown that mitochondrial function increases during preimplantation development especially starting from the activation of the embryonic genome to support blastocyst formation, which requires high levels of ATP [3,4]. Furthermore, mitochondrial function is known to decline with maternal age in oocytes and human embryos in the absence of effects on mitochondrial DNA (mtDNA) copy number. This determines the reduction of the kinetics and the rate of development to blastocysts [5]. At the convergence of cellular catabolic and anabolic pathways, mitochondria act as a hub in cellular signaling, energetics, and redox balances [6]. As a result of the energy-supplying function of mitochondria, reactive oxygen species (ROS) are produced. Originally considered an unavoidable by-product of oxidative phosphorylation, ROS have become known as crucial signaling molecules when their levels are kept within physiological range [6].

Several elements are essential for mitochondrial functioning, either by acting as cofactors in energy metabolism and/or antioxidants. These two functions are linked, as the antioxidant function may prevent damage to enzymes involved in energy metabolism, thereby limiting the reduction in energy production [7]. In this regard, there has been an increasing appreciation of the role of carnitines as mitochondrial modulators in oocytes and embryos. The main physiological properties of carnitines are regulation of the transport of acyl-CoA to the mitochondria matrix, the rate-limiting step of fatty acid oxidation, storage of energy as acetyl-carnitine, the modulation of the toxic effects of poorly metabolized acyl groups, and antioxidant activity [8,9]. In addition, the finding that low carnitine level is associated with metabolic diseases and aging has opened new frontiers in the use of carnitines as diet micronutrients that may improve fertility potential in patients suffering from obesity, diabetes, and polycystic ovary syndrome (PCOS) [9,10]. Therefore, the regulatory role of carnitine in mitochondrial energetic homeostasis and redox balance in oocytes and early embryos deserves proper attention.

In this context, the present review aimed to summarize current knowledge on the biological role of carnitines as modulators of mitochondrial metabolic activity, to provide the basis for targeted in vitro and in vivo use of carnitines to sustain and restore oocyte competence and female fertility

## 2. Mitochondrial Bioenergetics in Oocyte and Early Embryos

Metabolic activities that take place in the mitochondria provide most of the energetic supply of oocytes and early embryos. Metaphase II stage oocytes possess between 150,000 and 300,000 mitochondria, a condition indicative of high energetic cell demand [11,12]. Unlike somatic cells, oocyte mitochondria are generally more rounded and with few cristae, a condition that reflects the low level of electron transport chain (ETC) activity in order to efficiently meet energy demands while reducing the production of ROS, associated with oxidative phosphorylation [11,13]. In oocytes, this phenomenon probably underlies the logic behind the ‘quiet embryo hypothesis’ or ‘Goldilocks principle’ described by Leese et al. [14], proposing that the viability of early embryos is dependent upon their ability to maintain an optimal level of metabolism. Dysfunctional oocyte mitochondria are a major factor in the predisposition to aberrant development of embryos in female reproductive aging [11,12,15]. Fine regulation of mitochondrial activity and redox status is particularly important, since oocytes are highly dependent on oxidative phosphorylation for ATP generation, given the lack of expression of the high-affinity insulin-regulated glucose transporter SLC2A4 (or GLUT4) and low activity of the rate-limiting glycolysis enzyme phosphofructokinase [16,17]. Pyruvate and lactate for mitochondrial oxidative phosphorylation are provided by cumulus cells and enter the mitochondrial matrix via monocarboxylate transporters (MCTs) to be processed by the pyruvate dehydrogenase (PDH) complex to create acetyl CoA [18,19]. Pyruvate in in vitro culture media is taken up into the oocyte by MCTs. Diminished pyruvate inhibits oocyte activation and reduces developmental potential [20,21]. Given its ability to scavenge hydrogen peroxide, low pyruvate level negatively affects not only ATP production but also oocyte redox state [22].

**Figure 1:**
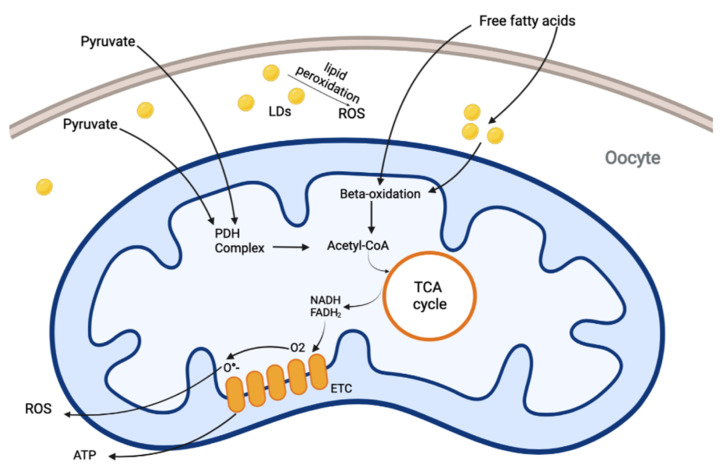
Mitochondrial pyruvate and fatty acid metabolism. Pyruvate from cumulus cells, follicular fluid, or culture medium enter the oocyte and is converted to acetyl-CoA by PDH complex. Free fatty acids are taken up into the oocyte to form an endogenous store in the form of lipid droplets (LDs). It is also possible that free fatty acids enter the mitochondria to undergo beta-oxidation. TCA cycle consumes acetyl-CoA and reduces NAD^+^ to NADH and FAD to FADH_2_. These factors are than oxidized by the electron transport chain to generate ATP through oxidative phosphorylation. The ETC can allow leakage of electrons back into the mitochondrial matrix, producing the superoxide radical (O_2_^−^). In the case of excess lipid intake, free fatty acids remain in the cytoplasm where they can generate lipid peroxides. A fine regulation of pyruvate and fatty acids metabolism is required to maintain energetic homeostasis, avoiding excess ROS production with negative effects on oocytes.

Despite a clear requirement for pyruvate metabolism for successful oocyte development, in recent years it has been recognized the importance of lipid metabolism [23]. Lipids are main mitochondrial fuels; however, these molecules can also behave as uncouplers and inhibitors of oxidative phosphorylation. Indeed, healthy mammalian oocyte mitochondria require a balance of pyruvate and fatty acid oxidation to maintain a low level of damaging ROS production. This balance can be disrupted in conditions of excess or insufficient substrate (Figure 1).

One of the important processes of lipid metabolism is beta-oxidation of fatty acids. Free fatty acids within the blood serum enter the follicular fluid, which surrounds the developing oocyte and cumulus cells and are taken up into the oocyte. A thorough evaluation of fatty acid composition in the follicular fluid revealed that esterified (triglycerides, cholesterol esters, and phospholipids) and non-esterified fatty acids are present. Palmitic acid (PA) and oleic acid (OA) are the two predominant saturated and unsaturated fatty acids, respectively, in mammalian serum and follicular fluid [24]. In mammalian oocytes, the greatest fraction of lipids is saturated fatty acids, mainly represented by palmitic and stearic acids [25]. Maternal diet can have a profound effect on the fatty acid composition of the follicular fluid, which may subsequently affect the fatty acid composition of the cumulus cells and the oocyte [26,27]. Among them, only triglycerides and non-esterified fatty acids were found to be affected by body mass index (BMI) of in vitro fertilization (IVF) patients [28].

Fatty acids are stored as neutral lipids in lipid droplets (LDs). A large number of them are actively esterified to a glycerol molecule catalyzed by the diacylglycerol acyltransferase (DGAT) and stored as neutral triacylglycerols (TAGs), which form a core surrounded by a single phospholipid layer, often with various protein inclusions [29,30]. Degradation of droplets tends to occur via lipolysis of TAGs to fatty acyl-CoA by lipases at the LDs’ surface, in order to make fatty acids available for mitochondrial metabolism.

Most of the enzymes involved in beta-oxidation are expressed in the human oocyte [31]. AcylCoA synthetase (ACS), the enzyme required for the activation of the fatty acid chains, is the most expressed in cumulus cells and oocytes [31]. Declined energy production and mitochondrial dysfunctions that occur during reproductive aging may be related to beta-oxidation as suggested by the finding that beta-hydroxyacyl-CoA dehydrogenase and 3-ketoacyl-CoA thiolase activity decrease in the aged oocyte [32]. An increased availability of fatty acids may lead to an increase in their mitochondrial oxidation. Igosheva et al. [33] found that mothers on a high fat diet have an increased oviductal leptin, which stimulates upregulation of genes controlling fatty acid oxidation, through activation of the nuclear PPAR-gamma receptors by altering mitochondrial functions in mouse oocytes and zygotes. It has also observed that exposure to high concentrations of palmitic acid reduces the redox state of oocytes, probably because of an increased activity of the tricarboxylic acid cycle (TCA) and the production of ROS, especially in cases where oxidative phosphorylation cannot keep up with the extent of substrate oxidation, and electron leakage ensues [34]. On the other hand, incomplete beta-oxidation would result in the accumulation of shorter acyl chains in the cytoplasm, which is susceptible to peroxidation, leading to lipotoxicity [35]. Lipotoxicity caused by excessive lipid accumulation and increased intracellular free fatty acid content leads to mitochondrial dysfunction, which can compromise mitochondrial function through free fatty acid beta-oxidation and oxidative stress, with dramatic effects on endoplasmic reticulum [36]. In vitro induced lipotoxicity in mouse oocytes has been associated with endoplasmic reticulum stress rather than mitochondrial dysfunction [37].

The use of a mitochondrial modulator which promotes fatty acid uptake such as l-carnitine is shown to rescue mitochondria from lipotoxic stress in numerous cell systems, while inhibition of fatty acid uptake increases cell death [38,39]. Accordingly, forced-lipophagy promotes survival of cleavage-stage embryos exposed to excess lipid, demonstrating that maintenance of the proper amounts of LDs is important for early embryonic development [40]. On the other hand, underfeeding and low body weight are also associated with low blastocyst rates and increased apoptosis in blastocyst cells, suggesting the relevance of a fine regulation of energetic requirement for successful oocyte development [41,42].

Studies using pharmacological inhibitors have confirmed the essential role of beta-oxidation in both oocyte nuclear maturation and the acquisition of developmental competence. Beta-oxidation is required for the resumption of meiosis and nuclear maturation in the mouse, bovine, and porcine oocytes [43,44,45]. Importantly, beta-oxidation increased in cumulus-enclosed oocytes that were stimulated to undergo maturation in vitro [23]. Using a functional beta-oxidation assay, Dunning et al. [23] demonstrated that the oocyte metabolizes about 15% of palmitic acid consumed in whole cumulus cell–oocyte complexes (COCs). Mouse COCs matured in vitro metabolize fatty acid at less than half the rate of COCs matured in vivo, in association with dysregulated expression of at least 15 genes involved in fatty acid activation, transport, and oxidation [24]. Elevated levels of fatty acids within the oocyte, particularly saturated fatty acids, lead to decreased rates of fertilization and embryo development, and can cause irreversible damage to fetal growth and offspring [46,47]. Beta-oxidation occurs during all stages of pre-implantation embryo development and peaks at the blastocyst stage [23,48].

**Figure 2:**
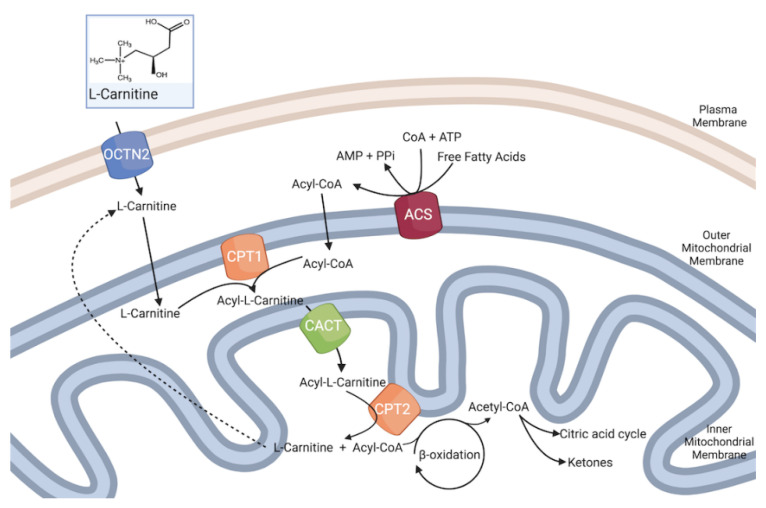
The carnitine shuttle. Long-chain fatty acids are converted to fatty acyl-CoAs by the enzyme acyl-CoA synthase (ACS), which is subsequently converted to fatty acylcarnitine by the enzyme carnitine palmitoyltransferase 1 (CPT 1), localized in the outer mitochondrial membrane. Fatty acylcarnitine is transported across the inner mitochondrial membrane via carnitine/acylcarnitine translocase (CACT) in exchange for l-carnitine. In the inner mitochondrial membrane, the enzyme carnitine palmitoyltransferase 2 (CPT 2) converts fatty acylcarnitine back to fatty acyl-CoAs and free l-carnitine, which exits the mitochondria and serves as the substrate for CPT 1 to form more acylcarnitine. These acetyl-CoA carbons then enter the citric acid cycle or are converted to ketones.

Fatty acid oxidation is tightly regulated at several points of the pathway to achieve a balance between energy production and expenditure. The rate-limiting step of fatty acid oxidation is the transport of fatty acyl-CoA to the mitochondria matrix through the carnitine system. This is important because acyl-CoA formed on the cytosolic side of mitochondria can either be directed to lipid biosynthesis or fatty acid oxidation (Figure 2).

## 3. Carnitines: Sources, Function, and Mechanism of Action

In mammals, the endogenous carnitine pool includes the un-esterified l-carnitine (l-3-hydroxy-4-*N*,*N*,*N*-trimethylaminobutyrate, LC) and many carnitine esters (acylcarnitines) with long- (i.e., palmitoyl-l-carnitine), medium- (i.e., octanoyl-l-carnitine), and short-chain (acetyl-l-carnitine, ALC and propionyl-l-carnitine, PLC). Acylcarnitines comprise a family of ~40 esters, of which ALC is the most abundant.

l-carnitine is synthesized endogenously, starting from the essential amino acids lysine and methionine at an approximate rate of 1 to 2 μmol/kg body weight/day in the liver, kidney, and brain, or assumed through intake of red meat, poultry, fish, and dairy products [49]. Dietary sources contribute to approximately 75% of the body store of LC, while the remaining 25% is formed endogenously. The combination of de novo biosynthesis, oral absorption, carrier-mediated distribution in tissues, and renal tubular reabsorption are responsible for maintaining carnitine homeostasis [49,50,51]. Carnitine is transported from the intestinal lumen into the enterocyte, with the fraction not absorbed in the small intestine being almost completely degraded by large intestine microbiota. The carnitine concentration in tissues is 20–50 times higher than in plasma (between 0.2–6 μmol/g). Carnitine is concentrated in high-energy-demanding tissues such as skeletal and cardiac muscles and in the epididymis. Muscle, liver, and kidney, and extracellular fluid compartments comprise 95.5, 3.5, and 1% of total body LC, respectively [9,52]. The concentration of LC in epididymal plasma and spermatozoa varies from 2 to 100 mM, which is nearly 2000-fold greater than circulating levels (10–50 μM) [53].

Carnitine has an obligatory role in the mitochondrial oxidation of long-chain fatty acids and the maintenance of mitochondrial functions. The oxidation of medium- (C6–C10) and short-chain (C4–C6) fatty acids seems largely independent from the so-called carnitine shuttle [54,55]. Carnitine takes part in the translocation of acetyl groups from the cytoplasm to the mitochondrion. Fatty acids derived from either de novo synthesis, dietary sources, or the turnover of TAG, phospholipids, and cholesterol esters have multiple metabolic fates. Fatty acids can also activate transcription factors, participate in intracellular signaling, and allosterically modulate enzyme reactions. Most of these functions require the formation of a long-chain acyl-CoA by acyl-CoA synthetases. Due to the lack of an acyl-CoA transporter in the mitochondrial inner membrane, the acyl group is transferred to the shuttle molecule carnitine for translocation into the matrix. Carnitine is accumulated inside the cell by the high-affinity organic cation transporter novel family member 2 (OCTN2)/solute carrier family 22 member 5 (SLC22A5). The expression of SLC22A5 is regulated by transcription factors, including peroxisome proliferator-activated receptors (PPARs) and estrogen receptor [56,57].

By reacting with acyl-CoA, carnitine forms a high-energy ester bond with long chain fatty acids by the action of carnitine palmitoyl transferase 1 (CPT-1), located in the outer mitochondrial membrane, with the formation of acyl-carnitines [58]. There are three isoforms of CPT-1: CPT-1A, expressed in the liver, brain, kidney, lung, spleen, intestine, pancreas, ovary, and fibroblasts; CPT-1B, the isoform highly expressed in heart, skeletal muscle, and testis; CPT1-C, the neuron specific isoform [54,59]. Acyl-carnitines are then translocated across the inner mitochondrial membrane by the carnitine acylcarnitine translocase (CACT, encoded by SLC25A20 gene) [58]. Once inside mitochondria, carnitine palmitoyl transferase 2 (CPT-2), located in the inner mitochondrial membrane, removes carnitine from acyl-carnitines and re-generates acyl-CoA [60]. Carnitine then returns to the cytoplasm for another cycle (using CACT), while the acyl-CoA (in aerobic conditions and in the presence of low levels of ATP) can enter beta-oxidation with final production of acetyl-CoA that enters the TCA. NADH and FADH2, generated by beta-oxidation and TCA, are the electron donors for the ETC.

Additional functions of the carnitine system have been described, including buffering of the acyl-CoA/CoA ratio, such that altered intermediary metabolism, disturbing free CoA/acyl-CoA homeostasis, is reflected in acylcarnitine concentrations. Therefore, the composition of the endogenous carnitine pool based on the evaluation of LC and acyl-carnitines is critical to characterize mitochondrial dysfunctions. Other roles for carnitines include branched-chain amino acid metabolism and peroxisomal fatty acid oxidation [61]. LC regulates the activity of several mitochondrial enzymes participating in mitochondrial metabolism [62,63,64]. In addition, carnitines appear to have anti-inflammatory and antioxidant properties, which include both the scavenger function against free radicals and the ability to chelate metal ions [8,65,66,67]. Moreover, carnitine can upregulate the transcription of catalase. Mitochondrial ALC is an important source of acetyl groups for histone acetylation, therefore contributing to transcription control [68]. Carnitine is also an activator of carbohydrate metabolism by promoting pyruvate oxidation, which is associated with the decrease in acetyl-CoA content [9]. Indeed, carnitine seems to improve insulin sensitivity [69,70].

Some evidence suggests that ALC and especially PLC have greater therapeutic effects than LC, probably due to the different pharmacokinetics of these acylcarnitines [71]. PLC, however, is not just a metabolic drug but also a powerful anti-radical agent. When PLC is administered exogenously, due to its high lipophilicity and affinity for acetyl-carnitine transferase, it is rapidly converted to free LC and propionyl-CoA. In these two forms, PLC plays an important role in both carbohydrate and lipid metabolism, leading to an increase in ATP efflux [72]. Interestingly, PLC offers some potentially advantageous properties over LC such as the ability to replenish intermediates of the TCA by the propionyl-CoA moiety. Overall, PLC is more effective than an equimolar dose of LC in promoting a higher level of energy expenditure [72]. It has also been suggested that PLC can be used directly by cells before being hydrolyzed to propionyl-CoA and LC. Indeed, PLC has been shown to protect tissues from oxidative damage and stabilize biological membranes by affecting their molecular dynamics and phospholipid turnover [72].

## 4. Carnitine Deficiency

Carnitine deficiency can be defined as a reduction of intracellular carnitine levels, which induce an uptake of acyl-CoA esters and an inhibition of acyl-transport via the mitochondrial inner membrane [73]. The primary carnitine deficiency is a rare autosomal recessive disorder of the carnitine cycle, caused by homozygous or heterozygous mutations in the SLC22A5 gene, encoding the OCTN2 carnitine transporter [58,74]. The lack of OCTN2 can determine high renal loss of carnitine, lower concentrations of carnitine in blood and tissues, and decreased intracellular carnitine accumulation [58,74]. The consequences of carnitine deficiency can differ depending on the age of onset [75,76]. A low carnitine level of the neonate can reflect a significant decrease in the plasma concentrations of total and free carnitine during pregnancy in healthy women, as carnitine can be transported through the placenta [77,78].

Recent studies reported that serum concentration of total carnitines was reduced in PCOS patients [79]. By contrast, Celik et al. [80] observed that when PCOS patients were compared with controls with the same BMI, it emerged that carnitine deficiency was linked to the obese phenotype and not to PCOS. This finding is in accordance with the observation that obesity results in reduced plasma carnitine [81]. Noteworthy, a recent study focusing on ovarian follicle metabolism observed that granulosa cells isolated from overweight/obese IVF patients showed higher levels of utilization of medium-chain fatty acid octanoyl-l-carnitine and higher mitochondrial metabolic activity [82]. Interestingly, this is in accordance with a previous metabolomic study on follicular fluid that has confirmed that levels of some carnitines are altered in PCOS women. These may account for compromised mitochondrial function and low ATP production as evidence of altered follicular microenvironment and oocyte competence in PCOS [83].

Most of literature reveals that plasma free carnitine levels are reduced in Type 1 and type 2 diabetic patients [84,85]. Since accumulation of acetyl-CoA is related to development of insulin resistance, carnitine supplementation has gained attention as a tool to treat insulin resistance and Type 2 diabetes [72].

Metabolome-wide association study revealed that plasma concentrations of long-chain and very long-chain carnitine increase with age, whereas odd chain carnitine levels, such as PLC, decrease [86]. Therefore PLC, one of the most powerful carnitines, may deserve attention in the context of anti-aging strategies.

## 5. Carnitine and Female Reproductive System

### 5.1. Carnitine Metabolism in the Oocytes and Embryos

Carnitine metabolism in the oocyte and embryos has been so far very poorly investigated. The only study so far was that by Montjean et al. [31], who observed that transcripts of two genes of the carnitine synthesis pathway (*N*6-trimethyllysine hydrolase and gamma-butyrobetaine hydroxylase) were expressed at low levels in human oocytes. The absence of expression of enzymes involved in carnitine synthesis in cumulus cells demonstrates the oocyte dependence on carnitine uptake from follicular fluid, where carnitine was found to range from 0.6 to 45.8 μmol/L with no correlation to IVF outcomes [31].

When carnitine shuttle system was investigated, the expression of carnitine acyl transferase, carnitine-acylcarnitine translocase, and carnitine acyltransferase II were found to be expressed in the oocyte, but transferases were poorly expressed in cumulus cells (CCs) [31]. In the mouse, Cpt1b transcripts were first and temporally detected at the two-cell stage and reappeared at the morula and blastocyst stage [87]. Cpt2 transcripts decreased following fertilization to undetectable levels and were present again later at the morula stage concomitantly with oxygen uptake and fatty acids oxidation [87]. Moreover, a correlation between the timing of fatty acids oxidation during preimplantation development and the expression of two genes encoding two enzymes involved in the oxidative pathway was found [87]. Furthermore, data obtained suggest a possible correlation between the levels of Cpt2 expression and embryo developmental competence [87].

### 5.2. The Potential of Carnitine Supplementation to Improve Female Fertility

Numerous lines of evidence indicate that LC and its acetylated form, ALC, possess excellent functional capabilities in the regulation of the metabolic and oxidative state of the female reproductive system.

#### 5.2.1. In Vitro Studies

Considering that LC is a beta-oxidation cofactor absent in most culture media, numerous in vitro studies have been carried out in order to investigate whether the oocyte competence and embryonic development enhanced in the presence of LC or ALC. Positive effects of LC supplementation to either oocyte maturation or embryo culture media have been demonstrated in several studies, including the improvement in in vitro maturation (IVM) rate, embryo development, and survival after cryopreservation. Many studies have been carried out in different mammalian species at various carnitine concentrations. A few of them have focused only on the analysis of functional parameters. In camels, LC supplementation with 0.5 mg/mL increased nuclear maturation and the number of morula and blastocysts [88]. LC supplementation during maturation improved the development of bovine embryos from meiotically less competent oocytes and accelerated blastocyst formation from more competent oocytes [89]. Carrillo-González et al. [90] discovered that the LC supplementation, at the concentrations of 3.8 and 1.5 mM in culture media, enhanced the pregnancy rate of in vitro produced embryos in the bovine model. Human embryos exposed to LC after fertilization exhibited improved embryo quality and were associated with higher implantation, as well as clinical and ongoing pregnancy [91].

**Figure 3:**
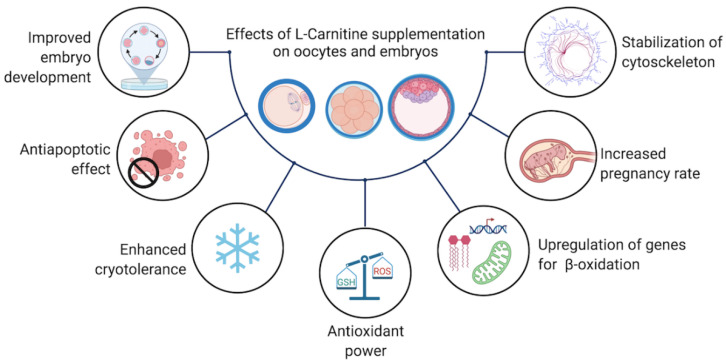
Effects of l-carnitine supplementation in media for oocyte and embryo culture.

Numerous in vitro studies have reported the mechanisms through which carnitines exert positive effects on development of oocytes and embryos; underlying mechanisms include beta-oxidation, antioxidant power, and protection against apoptosis (Figure 3).

##### Effects on Beta-Oxidation

Numerous studies revealed that carnitine addition to culture media improves lipid utilization with positive effects on energetic metabolism and oocyte and embryo competence. LC supplementation throughout in vitro 3D follicle culture significantly increased beta-oxidation in the follicle and have beneficial effects on the maturation and fertilization rate of oocytes and increased the number of blastocysts [92]. Mouse zygotes cultured in the absence of carbohydrates did not cleave, but supplementation with LC increased dose-dependently the number of zygotes cleaving to the two-cell stage, demonstrating the contribution of carnitine to embryo energetic metabolism [92]. Addition of LC to embryo culture media also improved embryo growth and this effect seems to be specifically due to the utilization of lipids. LC supplementation (5 mM) increased development of bovine embryos from zygote to the morula stage in the absence of carbohydrates, an effect that did not occur in the presence of the beta-oxidation inhibitor etomoxir [93]. Lowe et al. [94] described that oocyte exposure to 3 mM of LC prior to insemination enhanced cleavage and improved the cryotolerance of resulting blastocysts. This effect was probably due to LC-mediated reduction of oocyte lipid content. Accordingly, the supplementation with LC in culture media during in vitro bovine embryo production can improve post-thaw cryotolerance [95].

##### Antiapoptotic Effects

LC supplementation (0.5 mg/mL) of porcine COCs during IVM improved nuclear maturation, and, following parthenogenic activation, resulted in more blastocysts with fewer apoptotic cells, revealing that beneficial effects of LC may be related to its anti-apoptotic effect [96]. This observation was confirmed in studies performed in a mouse model. Indeed, when mouse embryos are treated with hydrogen peroxide or tumor necrosis factor (TNF), blastocyst development rates decrease in association with increased apoptosis. Nevertheless, the administration of 0.3 or 0.6 mg/mL of LC significantly reversed the adverse effects of both hydrogen peroxide and TNF [67].

In another model of embryo stress, the incubation of mouse eight-cell embryos with peritoneal fluid from patients with endometriosis significantly increased embryo apoptosis when compared with controls. Incubation with LC (0.6 mg/mL) improved microtubule and chromosome structure and decreased the level of embryo apoptosis [97]. An antiapoptotic and proliferative action of LC during embryo development was supported by the finding of alteration in the panel of apoptotic genes, including Bax, Bcl2, Casp3, and PCNA, and was confirmed by the observation that LC was able to counteract the proapoptotic and antiproliferative effects of actinomycin D and TNF-alpha on cleavage embryos [98].

Sheep oocytes matured with ALC showed significantly higher cleavage, morula, and blastocyst rate when compared with zygotes cultured with LC during post-fertilization period [99]. Supplementation of this acetyl-carnitine during IVM improved buffalo oocyte quality after vitrification by enhancing mitochondrial function and altering the phospholipid composition of vitrified oocyte membranes [100]. By carrying out TUNEL assay, it was found that an antiapoptotic mechanism underlies the beneficial effects of LC on follicular survival and ovarian function following cryopreservation–thawing and autotransplantation of ovarian tissues [101]. Antiapoptotic effects of LC in terms of increased Bcl2/Bax ratio have been associated with the recovery of ovarian function following chemotherapy in rats [102].

##### Antioxidant Effects

Porcine oocytes matured in 1.25 mg/mL LC had significantly more active mitochondria, fewer LDs, and decreased ROS levels when compared to controls [103]. In other work, LC supplementation (0.5 mg/mL) of porcine COCs during IVM reduced oocyte ROS levels and increased production of the antioxidant glutathione (GSH) during IVM [96].

In the mouse, 0.3 or 0.6 mg/mL LC significantly reversed the damaging effects of hydrogen peroxide on embryos cleavage from the eight-cell to blastocyst stage [67]. The antioxidant effect of LC was confirmed by the observation that carnitine supplementation was able to protect mouse oocytes and embryos from H_2_O_2_-induced oxidative damage. Indeed, adding 0.6 mg/mL LC into maturation medium of immature oocyte decreased ROS level via a scavenging effect on 1,1-diphenyl-2-picryl-hydrazyl free radical (DPPH), superoxide anion radical, and hydrogen peroxide [104]. Treating embryos developed from in vitro aged bovine oocytes with LC results in reduced ROS, increased GSH level and expression of Sod1 and Gpx4, increased mitochondrial membrane potential, and autophagy with subsequent improvement of blastocyst rate and quality [105].

You et al. [106] demonstrated that LC treatment during IVM improves the developmental competence of SCNT embryos. This effect is probably due to increased intracellular GSH synthesis in recipient ooplasts, which reduces ROS levels, and the stimulation of nuclear reprogramming via increased expression of POU5F1 and transcription factors.

Two different studies investigated the potential use of LC as a scavenger for ROS contained in peritoneal and follicular from patients with endometriosis. Endometriosis is a common gynecological disorder characterized by ovarian, peritoneal, as well as deep infiltrating endometriotic lesions [97,107]. Studies on endometriosis reveal the presence of oxidative stress and inflammatory factors that alter the abdominal microenvironment and may contribute to infertility. In 2009, Mansour et al. [97] exposed mouse oocytes and embryos to peritoneal fluid from patients with endometriosis and demonstrated that the presence of LC (0.6 mg/mL) prevented the occurrence of spindle and chromosome changes in oocytes and of apoptosis in blastocysts. The authors suggest that these effects may be ascribed to LC antioxidant activity, probably acting by modulating cytokines, such as TNF-alpha, known to be present in the peritoneal fluid from endometriosis patients [97]. Similar improvements of spindle instability were obtained when bovine oocytes were cultured in vitro in follicular fluids from patients with endometriosis. LC (0.6 mg/mL) addition during in vitro culture was able to counteract meiotic errors and was more effective than *N*-acetyl cysteine (NAC) or combination of LC and NAC [108]. By contrast, LC did not improve embryo development of bovine zygotes cultured in follicular fluids from infertile women with mild endometriosis, as occurs with NAC supplementation [109]. In summary, these observations suggest that LC may contribute to counteract oocyte alterations by restoring faulty energy metabolism of fatty acids rather than by modulating antioxidant activity.

##### Effects on Mitochondria

There is evidence that LC supplementation (0.02% for 14 days) during in vitro culture of porcine early antral follicles and maturation of oocytes increases the number of MII oocytes via increased mitochondrial activity, as assessed by MitoTracker Red CMXRos [103].

Xu et al. [100] investigated whether oocyte quality and embryonic development enhanced with the addition of ALC (2.5 mM) during in vitro maturation of buffalo oocytes. From this study it emerged that ALC ameliorated the quality of matured buffalo oocytes and the embryonic development in vitro acting on mitochondrial function, regulating oocyte-derived paracrine factors, and increasing the production of steroid hormones [100]. This suggests that ALC may have the same function as LC in promoting mitochondrial activity in MII oocytes after vitrification [110], indicating that ALC supplementation during IVM improves the cryotolerance of mature buffalo oocytes by increasing mitochondrial membrane potential after vitrification.

Supplementation of culture media with LC significantly improves mitochondrial function in human embryos at the morula stage by increasing oxygen consumption rate and ATP production, with no effects on mitochondrial copy numbers [111].

#### 5.2.2. In Vivo Studies

Different studies reported the effects of carnitines’ administration on fertility. In vivo, circulating carnitine levels are normally regulated within a narrow range, and thus dietary carnitine supplementation may only affect tissue levels when carnitine levels are systemically dysregulated. In contrast to the multiple reports demonstrating that LC treatment in vitro can improve embryo development, little is known about whether circulating carnitine or in vivo supplementation with LC can improve oocyte quality.

In a mouse model of repeated ovulation cycles, oral administration of LC (5 mg/mL) improved the number of oocytes ovulated and their competence in terms of mitochondrial mass and distribution, and oxidative damage in the oocyte and ovary. A reduction of oocyte NRF1 (nuclear respiratory factor 1) and 8-OHdG (8-hydroxy-2′-deoxyguanosine) was observed along with potentiation of mitochondrial superoxide dismutase (SOD2) in the ovarian microenvironment [112].

The only reproductive dysfunction known to be associated with carnitine deficiency is PCOS. PCOS is a frequent reproductive disorder that affects approximately 4–25% of women of reproductive age. It is diagnosed by the observation of oligomenorrhea and/or anovulation, clinical and/or biochemical signs of hyperandrogenism, and polycystic echo structure of the ovary, which are usually accompanied with hyperinsulinemia and insulin resistance. A lean and an overweight/obese phenotype are reported. As described above, serum concentration of total LC was found to be reduced in PCOS patients, with a more pronounced effect in obese PCOS women [79,80]. Different reports have identified that diet supplementation with LC enhances the efficacy of common PCOS therapies by improving biochemical, endocrine, and anthropometric parameters [113,114,115,116]. Indeed, Samimi et al. [114] observed that at the end of 12 weeks of carnitine administration (250 mg/day) in PCOS women, there occurred a reduction in weight, BMI (body mass index), waist circumference, and hip circumference, as well as beneficial effects on glycemic control. In a study on women with clomiphene-resistant PCOS, Ismail et al. [113] reported that the addition of LC to therapy with clomiphene citrate enhanced thickness of the endometrium, estradiol concentrations, the quality of ovulation, pregnancy rates, lipid profiles, and the BMI. A significant reduction in the BMI and serum levels of triglyceride, LDL cholesterol, fasting blood sugar, and insulin, in addition to increased serum HDL cholesterol levels, have been observed in a study in which the effects of LC on clinical and laboratory findings of patients with PCOS were evaluated [115]. Furthermore, El Sharkwy and El-Din [116] demonstrated that the co-treatment with LC and metformin in clomiphene citrate-resistant obese PCOS women acted synergically in the improvement of reproductive performance, insulin resistance, and lipid profile.

More recently, new formulations composed of different carnitine and other micronutrients have been administered to support therapies against PCOS. The use of a mix of ALC, LC, l-arginine, and NAC was found to reduce insulin resistance and improve insulin sensitivity and the hepatic insulin extraction index in overweight/obese PCOS patients [117]. When ALC was combined with metformin and pioglitazone, patients exhibit reduced menstrual irregularities in association with reduction of insulin in fasting blood samples, luteinizing hormone (LH), and body circumferences [118]. Two studies demonstrated that the co-administration of LC (1000 mg/day) and chromium (200 µg/day) for 12 weeks to women with PCOS had positive effects on weight loss, hirsutism, serum total testosterone, glycemic control, lipid profiles, high-sensitivity C-reactive protein, total antioxidant capacity, and lipid peroxidation levels [119,120]. Moreover, LC-chromium supplementation ameliorates inflammatory status, as confirmed by reduced levels of gene expression of IL-6 and TNF-alpha in lymphocyte [119].

In order to obtain a better comprehension of the molecular mechanisms underlying LC activity on ovarian microenvironment and oocyte competence, LC was administered in different PCOS mouse models. Kalhori et al. [121] developed a PCOS mouse by testosterone enanthate, which presented anovulation, an increased number of apoptotic follicles, and hyperandrogenism. In these PCOS mice, LC administration improved the function and structure of the ovary through favorable changes in follicular development and FSH, LH, and testosterone. Interestingly, these authors found a reduction in inflammatory and pro-oxidant markers, such as IL-6, TNF-alpha, and malondialdehyde (MDA), which are considered as important factors affecting folliculogenesis in PCOS [121]. In a recent work from our research group, we developed two different carnitine formulations that were orally administered to a DHEA (dehydroepiandrosterone)-induced PCOS mouse model [122]. Unlike other studies on this mouse model, carnitine formulations were developed based on carnitine doses employed in dietary supplementation for human use [123]. The first formulation containing 0.40 mg LC and 0.20 mg ALC (mouse/day) was effective in ameliorating ovarian aspect and functionality and in increasing the number and quality of ovulated oocytes obtained after superovulation. These effects were observed also in PCOS mice receiving the second formulation containing 0.40 mg LC, 0.20 mg ALC, and 0.08 mg PLC (mouse/day), where a decrease in serum levels of testosterone was also reported. The study of molecular changes induced by carnitines highlighted that both carnitine formulations positively modulated the antioxidant and glycative adaptive response of PCOS mouse ovaries, regulated by SIRT1 [124], and promoted the potentiation of mitochondria by modulating the expression of the mitochondrial transcription factor A (mtTFA), SIRT3, and SOD2. Interestingly, the presence of PLC provides better activity on molecular and functional parameters that have been considered [123].

## 6. Conclusions

From the literature emerges a clear picture of the role of carnitines as modulators of the mitochondrial metabolic activity, which governs bioenergetic homeostasis in the reproductive processes driving oocyte competence and embryos’ ability to develop into a healthy newborn. In this regard, some carnitines are promising culture medium supplements that might be able to counteract altered energy and redox homeostasis, associated with in vitro culture and cryopreservation, with important clinical applications in the assisted reproduction setting. Furthermore, the literature provides relevant support to the potential use of carnitines as micronutrients exerting therapeutic effects on fertility dysfunctions in PCOS. Effects of carnitine on PCOS women and animal models include the restoration of metabolic parameters and inflammatory status, ovarian functionality, and oocyte competence. It is also important to consider that, except for the study by Genazzani et al. [117], who assessed a mix containing LC and ALC, human research has been carried out exclusively on LC. Of the two studies conducted on the animal PCOS model, one has tested LC and the other one has tested LC in combination with ALC. In addition, the latter is the only study which has taken into account and discovered the potentiality of PLC to boost the beneficial effects of LC and ALC, indicating that this acylcarnitine and its mechanisms of action deserve further investigation. Pathways activated by carnitine exposure or administration include antiapoptotic, antioxidant, and antiinflammatory signaling. An effect against AGE formation (i.e., antiglycative effect) has also been revealed. Furthermore, oocyte, embryo, and ovarian mitochondrial functions are positively targeted by in vitro and in vivo carnitine supplementation. Nevertheless, the potential of carnitine to improve lipid utilization with positive effects on energetic metabolism and oocyte and embryo competence has been very poorly investigated. This may be explained by a reduced availability of methods for studying lipid metabolism in oocytes and embryos, as well as the scarce knowledge of the molecular machinery involved in carnitine regulation of mitochondrial metabolism in oocytes and embryos. This point warrants better investigation, considering that healthy mammalian oocyte mitochondria require a balance of pyruvate and fatty acid oxidation in order to maintain low levels of ROS production. Being aware that this balance is disrupted in conditions of excess or insufficient substrate, as occurs in the case of being overweight or underfeeding, carnitines may act as mitochondrial modulators, promoting the fine regulation of energetic requirement for successful oocyte development and pregnancy.

## References

[1] Fontana R., Della Torre S. (2016). The Deep Correlation between Energy Metabolism and Reproduction: A View on the Effects of Nutrition for Women Fertility. Nutrients.

[2] Warzych E., Lipinska P. (2020). Energy metabolism of follicular environment during oocyte growth and maturation. J. Reprod. Dev..

[3] Trimarchi J.R., Liu L., Porterfield D.M., Smith P.J., Keefe D.L. (2000). Oxidative Phosphorylation-Dependent and -Independent Oxygen Consumption by Individual Preimplantation Mouse Embryos. Biol. Reprod..

[4] Hashimoto S., Morimoto N., Yamanaka M., Matsumoto H., Yamochi T., Goto H., Inoue M., Nakaoka Y., Shibahara H., Morimoto Y. (2017). Quantitative and qualitative changes of mitochondria in human preimplantation embryos. J. Assist. Reprod. Genet..

[5] Morimoto N., Hashimoto S., Yamanaka M., Nakano T., Satoh M., Nakaoka Y., Iwata H., Fukui A., Morimoto Y., Shibahara H. (2020). Mitochondrial oxygen consumption rate of human embryos declines with maternal age. J. Assist. Reprod. Genet..

[6] Venditti P., Di Meo S. (2020). The Role of Reactive Oxygen Species in the Life Cycle of the Mitochondrion. Int. J. Mol. Sci..

[7] Wesselink E., Koekkoek W.A.C., Grefte S., Witkamp R.F., van Zanten A.R.H. (2019). Feeding mitochondria: Potential role of nutritional components to improve critical illness convalescence. Clin. Nutr..

[8] Solarska-Ściuk K., Lewinska A., Karowicz-Bilińska A., Bartosz G. (2010). The antioxidant properties of carnitine in vitro. Cell. Mol. Biol. Lett..

[9] Pękala J., Patkowska-Sokola B., Bodkowski R., Jamroz D., Nowakowski P., Lochynski S., Librowski T. (2011). L-Carnitine-Metabolic Functions and Meaning in Humans Life. Curr. Drug Metab..

[10] Agarwal A., Sengupta P., Durairajanayagam D. (2018). Role of L-carnitine in female infertility. Reprod. Biol. Endocrinol..

[11] Eichenlaub-Ritter U., Wieczorek M., Lüke S., Seidel T. (2011). Age related changes in mitochondrial function and new approaches to study redox regulation in mammalian oocytes in response to age or maturation conditions. Mitochondrion.

[12] May-Panloup P., Boucret L., De La Barca J.-M.C., Desquiret-Dumas V., Ferré-L’Hotellier V., Morinière C., Descamps P., Procaccio V., Reynier P. (2016). Ovarian ageing: The role of mitochondria in oocytes and follicles. Hum. Reprod. Update.

[13] Dumollard R., Carroll J., Duchen M.R., Campbell K., Swann K. (2009). Mitochondrial function and redox state in mammalian embryos. Semin. Cell Dev. Biol..

[14] Leese H.J., Guerif F., Allgar V., Brison D.R., Lundin K., Sturmey R.G. (2016). Biological optimization, the Goldilocks principle, and how much is lagom in the preimplantation embryo. Mol. Reprod. Dev..

[15] Di Emidio G., Falone S., Artini P.G., Amicarelli F., D’alessandro A.M., Tatone C. (2021). Mitochondrial Sirtuins in Reproduction. Antioxidants.

[16] Cetica P., Pintos L., Dalvit G., Beconi M. (2002). Activity of key enzymes involved in glucose and triglyceride catabolism during bovine oocyte maturation in vitro. Reproduction.

[17] Sutton-McDowall M.L., Gilchrist R.B., Thompson J.G. (2010). The pivotal role of glucose metabolism in determining oocyte developmental competence. Reproduction.

[18] Donahue R.P., Stern S. (1968). Follicular Cell Support of Oocyte Maturation: Production of Pyruvate In Vitro. J. Reprod. Fertil..

[19] Leese H.J., Barton A.M. (1985). Production of pyruvate by isolated mouse cumulus cells. J. Exp. Zool..

[20] Dumollard R., Ward Z., Carroll J., Duchen M.R. (2007). Regulation of redox metabolism in the mouse oocyte and embryo. Development.

[21] Dumollard R., Campbell K., Halet G., Carroll J., Swann K. (2008). Regulation of cytosolic and mitochondrial ATP levels in mouse eggs and zygotes. Dev. Biol..

[22] Constantopoulos G., Barranger J.A. (1984). Nonenzymatic decarboxylation of pyruvate. Anal. Biochem..

[23] Dunning K.R., Cashman K., Russell D.L., Thompson J.G., Norman R.J., Robker R.L. (2010). Beta-Oxidation Is Essential for Mouse Oocyte Developmental Competence and Early Embryo Development. Biol. Reprod..

[24] Dunning K.R., Russell D., Robker R.L. (2014). Lipids and oocyte developmental competence: The role of fatty acids and β-oxidation. Reproduction.

[25] McEvoy T.G., Coull G.D., Broadbent P.J., Hutchinson J.S., Speake B.K. (2000). Fatty acid composition of lipids in immature cattle, pig and sheep oocytes with intact zona pellucida. J. Reprod. Fertil..

[26] Zachut M., Dekel I., Lehrer H., Arieli A., Arav A., Livshitz L., Yakoby S., Moallem U. (2010). Effects of dietary fats differing in n-6:n-3 ratio fed to high-yielding dairy cows on fatty acid composition of ovarian compartments, follicular status, and oocyte quality. J. Dairy Sci..

[27] Wonnacott K.E., Kwong W.Y., Hughes J., Salter A.M., Lea R.G., Garnsworthy P.C., Sinclair K.D. (2010). Dietary omega-3 and -6 polyunsaturated fatty acids affect the composition and development of sheep granulosa cells, oocytes and embryos. Reproduction.

[28] Valckx S.D., Arias-Alvarez M., De Pauw I., Fievez V., Vlaeminck B., Fransen E., Bols P.E., Leroy J.L. (2014). Fatty acid composition of the follicular fluid of normal weight, overweight and obese women undergoing assisted reproductive treatment: A descriptive cross-sectional study. Reprod. Biol. Endocrinol..

[29] Fujimoto T., Parton R.G. (2011). Not Just Fat: The Structure and Function of the Lipid Droplet. Cold Spring Harb. Perspect. Biol..

[30] Walther T.C., Farese R.V. (2012). Lipid Droplets and Cellular Lipid Metabolism. Annu. Rev. Biochem..

[31] Montjean D., Entezami F., Lichtblau I., Belloc S., Gurgan T., Menezo Y. (2012). Carnitine content in the follicular fluid and expression of the enzymes involved in beta oxidation in oocytes and cumulus cells. J. Assist. Reprod. Genet..

[32] Yazigi R.A., Chi M.M., Mastrogiannis D.S., Strickler R.C., Yang V.C., Lowry O.H. (1993). Enzyme activities and maturation in unstimulated and exogenous gonadotropin-stimulated human oocytes. Am. J. Physiol..

[33] Igosheva N., Abramov A.Y., Poston L., Eckert J.J., Fleming T.P., Duchen M.R., McConnell J. (2010). Maternal Diet-Induced Obesity Alters Mitochondrial Activity and Redox Status in Mouse Oocytes and Zygotes. PLoS ONE.

[34] Sutton-McDowall M.L., Wu L.L., Purdey M., Abell A.D., Goldys E.M., Macmillan K.L., Thompson J.G., Robker R.L. (2016). Nonesterified Fatty Acid-Induced Endoplasmic Reticulum Stress in Cattle Cumulus Oocyte Complexes Alters Cell Metabolism and Developmental Competence. Biol. Reprod..

[35] Muoio D.M., Neufer P.D. (2012). Lipid-Induced Mitochondrial Stress and Insulin Action in Muscle. Cell Metab..

[36] Hauck A.K., Bernlohr D.A. (2016). Oxidative stress and lipotoxicity. J. Lipid Res..

[37] Wang Y., Pope I., Brennan-Craddock H., Poole E., Langbein W., Borri P., Swann K. (2021). A primary effect of palmitic acid on mouse oocytes is the disruption of the structure of the endoplasmic reticulum. Reproduction.

[38] Oyanagi E., Yano H., Uchida M., Utsumi K., Sasaki J. (2011). Protective action of l-carnitine on cardiac mitochondrial function and structure against fatty acid stress. Biochem. Biophys. Res. Commun..

[39] Borradaile N.M., Han X., Harp J.D., Gale S.E., Ory D.S., Schaffer J.E. (2006). Disruption of endoplasmic reticulum structure and integrity in lipotoxic cell death. J. Lipid Res..

[40] Tatsumi T., Takayama K., Ishii S., Yamamoto A., Hara T., Minami N., Miyasaka N., Kubota T., Matsuura A., Itakura E. (2018). Forced lipophagy reveals that lipid droplets are required for early embryonic development in mouse. Development.

[41] Grazul-Bilska A.T., Borowczyk E., Bilski J.J., Reynolds L.P., Redmer D.A., Caton J.S., Vonnahme K.A. (2012). Overfeeding and underfeeding have detrimental effects on oocyte quality measured by in vitro fertilization and early embryonic development in sheep. Domest. Anim. Endocrinol..

[42] Kubandová J., Čikoš S., Burkuš J., Czikková S., Koppel J., Fabian D. (2014). Amount of maternal body fat significantly affected the quality of isolated mouse preimplantation embryos and slowed down their development. Theriogenology.

[43] Downs S.M., Mosey J.L., Klinger J. (2009). Fatty acid oxidation and meiotic resumption in mouse oocytes. Mol. Reprod. Dev..

[44] Paczkowski M., Silva E., Schoolcraft W.B., Krisher R.L. (2013). Comparative Importance of Fatty Acid Beta-Oxidation to Nuclear Maturation, Gene Expression, and Glucose Metabolism in Mouse, Bovine, and Porcine Cumulus Oocyte Complexes. Biol. Reprod..

[45] Valsangkar D., Downs S.M. (2013). A Requirement for Fatty Acid Oxidation in the Hormone-Induced Meiotic Maturation of Mouse Oocytes. Biol. Reprod..

[46] Jungheim E.S., Macones G.A., Odem R.R., Patterson B.W., Lanzendorf S.E., Ratts V.S., Moley K.H. (2011). Associations between free fatty acids, cumulus oocyte complex morphology and ovarian function during in vitro fertilization. Fertil. Steril..

[47] Luzzo K.M., Wang Q., Purcell S.H., Chi M., Jimenez P.T., Grindler N., Schedl T., Moley K.H. (2012). High Fat Diet Induced Developmental Defects in the Mouse: Oocyte Meiotic Aneuploidy and Fetal Growth Retardation/Brain Defects. PLoS ONE.

[48] Hillman N., Flynn T.J. (1980). The metabolism of exogenous fatty acids by preimplantation mouse embryos developing in vitro. J. Embryol. Exp. Morphol..

[49] Longo N., Frigeni M., Pasquali M. (2016). Carnitine transport and fatty acid oxidation. Biochim. Biophys. Acta.

[50] Rebouche C.J. (2004). Kinetics, Pharmacokinetics, and Regulation of L-carnitine and Acetyl-l-carnitine Metabolism. Ann. N. Y. Acad. Sci..

[51] Reuter S.E., Evans A.M. (2012). Carnitine and acylcarnitines: Pharmacokinetic, pharmacological and clinical aspects. Clin. Pharmacokinet..

[52] Evans A.M., Fornasini G. (2003). Pharmacokinetics of L-carnitine. Clin. Pharmacokinet..

[53] Brooks D.E., Frenkel R., McGarry J. (1980). Carnitine in the male reproductive tract and its relation to the metabolism of the epididymis and spermatozoa. Carnitine Biosynthesis Metabolism and Function.

[54] Schrader M., Costello J., Godinho L.F., Islinger M. (2015). Peroxisome-mitochondria interplay and disease. J. Inherit. Metab. Dis..

[55] Violante S., Ijlst L., Brinke H.T., Koster J., de Almeida I.T., Wanders R.J., Ventura F.V., Houten S.M. (2013). Peroxisomes contribute to the acylcarnitine production when the carnitine shuttle is deficient. Biochim. Biophys. Acta.

[56] Indiveri C., Iacobazzi V., Tonazzi A., Giangregorio N., Infantino V., Convertini P., Console L., Palmieri F. (2011). The mitochondrial carnitine/acylcarnitine carrier: Function, structure and physiopathology. Mol. Asp. Med..

[57] Casals N., Zammit V., Herrero L., Fadó R., Rodriguez-Rodriguez R., Serra D. (2016). Carnitine palmitoyltransferase 1C: From cognition to cancer. Prog. Lipid Res..

[58] Longo N., di San Filippo C.A., Pasquali M. (2006). Disorders of carnitine transport and the carnitine cycle. Am. J. Med. Genet. Part C Semin. Med. Genet..

[59] Lee N.-Y., Choi H.-O., Kang Y.-S. (2012). The Acetylcholinesterase Inhibitors Competitively Inhibited an Acetyl L-carnitine Transport through the Blood-Brain Barrier. Neurochem. Res..

[60] Houten S.M., Wanders R.J. (2010). A general introduction to the biochemistry of mitochondrial fatty acid β-oxidation. J. Inherit. Metab. Dis..

[61] Hoppel C. (2003). The role of carnitine in normal and altered fatty acid metabolism. Am. J. Kidney Dis..

[62] Rospond B., Chłopicka J. (2013). Funkcje biologiczne L-karnityny i jej zawartość w wybranych produktach spozywczych [The bio-logical function of L-carnitine and its content in the particular food examples]. Prz. Lek..

[63] Pietrocola F., Galluzzi L., Bravo-San Pedro J.M., Madeo F., Kroemer G. (2015). Acetyl Coenzyme A: A Central Metabolite and Second Messenger. Cell Metab..

[64] Almannai M., Alfadhel M., El-Hattab A.W. (2019). Carnitine Inborn Errors of Metabolism. Molecules.

[65] Ribas G.S., Vargas C.R., Wajner M. (2014). L-carnitine supplementation as a potential antioxidant therapy for inherited neurometabolic disorders. Gene.

[66] Lee B.-J., Lin J.-S., Lin Y.-C., Lin P.-T. (2014). Effects of L-carnitine supplementation on oxidative stress and antioxidant enzymes activities in patients with coronary artery disease: A randomized, placebo-controlled trial. Nutr. J..

[67] Abdelrazik H., Sharma R., Mahfouz R.Z., Agarwal A. (2009). L-carnitine decreases DNA damage and improves the in vitro blastocyst development rate in mouse embryos. Fertil. Steril..

[68] Madiraju P., Pande S.V., Prentki M., Madiraju S.M. (2009). Mitochondrial acetylcarnitine provides acetyl groups for nuclear histone acetylation. Epigenetics.

[69] Xu Y., Jiang W., Chen G., Zhu W., Ding W., Ge Z., Tan Y., Ma T., Cui G. (2017). L-carnitine treatment of insulin resistance: A systematic review and meta-analysis. Adv. Clin. Exp. Med..

[70] Bene J., Hadzsiev K., Melegh B. (2018). Role of carnitine and its derivatives in the development and management of type 2 diabetes. Nutr. Diabetes.

[71] Siliprandi N., Di Lisa F., Menabò R. (1991). Propionyl-L-carnitine: Biochemical significance and possible role in cardiac metabolism. Cardiovasc. Drugs Ther..

[72] Mingorance C., Rodriguez-Rodriguez R., Justo M.L., Herrera M.D., de Sotomayor M.A. (2011). Pharmacological effects and clinical applications of propionyl-L-carnitine. Nutr. Rev..

[73] El-Hattab A.W., Scaglia F. (2015). Disorders of carnitine biosynthesis and transport. Mol. Genet. Metab..

[74] Koepsell H. (2013). The SLC22 family with transporters of organic cations, anions and zwitterions. Mol. Asp. Med..

[75] Breningstall G.N. (1990). Carnitine deficiency syndromes. Pediatr. Neurol..

[76] Magoulas P.L., El-Hattab A.W. (2012). Systemic primary carnitine deficiency: An overview of clinical manifestations, diagnosis, and management. Orphanet J. Rare Dis..

[77] Lee N.-C., Tang N.L., Chien Y.-H., Chen C.-A., Lin S.-J., Chiu P.-C., Huang A.-C., Hwu W.-L. (2010). Diagnoses of newborns and mothers with carnitine uptake defects through newborn screening. Mol. Genet. Metab..

[78] Schoderbeck M., Auer B., Legenstein E., Genger H., Sevelda P., Salzer H., Marz R., Lohninger A. (1995). Pregnancy-related changes of carnitine and acylcarnitine concentrations of plasma and erythrocytes. J. Périnat. Med..

[79] Fenkci S.M., Fenkci V., Oztekin O., Rota S., Karagenc N. (2008). Serum total L-carnitine levels in non-obese women with polycystic ovary syndrome. Hum. Reprod..

[80] Celik F., Kose M., Yilmazer M., Köken G.N., Arioz D.T., Pektas M.K. (2017). Plasma L-carnitine levels of obese and non-obese polycystic ovary syndrome patients. J. Obstet. Gynaecol..

[81] Tipi-Akbas P., Arioz D.T., Kanat-Pektas M., Koken T., Koken G., Yilmazer M. (2013). Lowered serum total L-carnitine levels are associated with obesity at term pregnancy. J. Matern. Fetal Neonatal Med..

[82] Kordus R.J., Hossain A., Malter H.E., Lavoie H.A. (2020). Mitochondrial metabolic substrate utilization in granulosa cells reflects body mass index and total follicle stimulating hormone dosage in in vitro fertilization patients. J. Assist. Reprod. Genet..

[83] Zhao H., Zhao Y., Li T., Li M., Li J., Li R., Liu P., Yu Y., Qiao J. (2015). Metabolism alteration in follicular niche: The nexus among intermediary metabolism, mitochondrial function, and classic polycystic ovary syndrome. Free Radic. Biol. Med..

[84] Mamoulakis D., Galanakis E., Dionyssopoulou E., Evangeliou A., Sbyrakis S. (2004). Carnitine deficiency in children and adolescents with type 1 diabetes. J. Diabetes Complicat..

[85] Ringseis R., Keller J., Eder K. (2012). Role of carnitine in the regulation of glucose homeostasis and insulin sensitivity: Evidence from in vivo and in vitro studies with carnitine supplementation and carnitine deficiency. Eur. J. Nutr..

[86] Jarrell Z.R., Smith M.R., Hu X., Orr M., Liu K.H., Quyyumi A.A., Jones D.P., Go Y.-M. (2020). Plasma acylcarnitine levels increase with healthy aging. Aging.

[87] Gentile L., Monti M., Sebastiano V., Merico V., Nicolai R., Calvani M., Garagna S., Redi C.A., Zuccotti M. (2004). Single-cell quantitative RT-PCR analysis of Cpt1b and Cpt2 gene expression in mouse antral oocytes and in preimplantation embryos. Cytogenet. Genome Res..

[88] Fathi M., El-Shahat K.H. (2017). L-carnitine enhances oocyte maturation and improves in vitro development of embryos in dromedary camels (*Camelus dromedaries*). Theriogenology.

[89] Knitlova D., Hulinska P., Jeseta M., Hanzalova K., Kempisty B., Machatkova M. (2017). Supplementation of l-carnitine during in vitro maturation improves embryo development from less competent bovine oocytes. Theriogenology.

[90] Carrillo-González D.F., Maldonado-Estrada J.G. (2020). L-carnitine supplementation in culture media improves the pregnancy rate of in vitro produced embryos with sexed semen from Bos taurus indicus cows. Trop. Anim. Health Prod..

[91] Kim M.K., Park J.K., Paek S.K., Kim J.W., Kwak I.P., Lee H.J., Lyu S.W., Lee W.S. (2018). Effects and pregnancy outcomes of L-carnitine supplementation in culture media for human embryo development from in vitro fertilization. J. Obstet. Gynaecol. Res..

[92] Dunning K.R., Akison L.K., Russell D.L., Norman R.J., Robker R.L. (2011). Increased Beta-Oxidation and Improved Oocyte Developmental Competence in Response to L-carnitine during Ovarian In Vitro Follicle Development in Mice. Biol. Reprod..

[93] Sutton-McDowall M.L., Feil D., Robker R.L., Thompson J.G., Dunning K.R. (2012). Utilization of endogenous fatty acid stores for energy production in bovine preimplantation embryos. Theriogenology.

[94] Lowe J.L., Bartolac L.K., Bathgate R., Grupen C.G. (2017). Cryotolerance of porcine blastocysts is improved by treating in vitro matured oocytes with L-carnitine prior to fertilization. J. Reprod. Dev..

[95] Zolini A.M., Carrascal-Triana E., de King A.R., Hansen P.J., Torres C.A.A., Block J. (2019). Effect of addition of l-carnitine to media for oocyte maturation and embryo culture on development and cryotolerance of bovine embryos produced in vitro. Theriogenology.

[96] Wu G.-Q., Jia B.-Y., Li J.-J., Fu X.-W., Zhou G.-B., Hou Y.-P., Zhu S.-E. (2011). L-carnitine enhances oocyte maturation and development of parthenogenetic embryos in pigs. Theriogenology.

[97] Mansour G., Abdelrazik H., Sharma R.K., Radwan E., Falcone T., Agarwal A. (2009). L-carnitine supplementation reduces oocyte cytoskeleton damage and embryo apoptosis induced by incubation in peritoneal fluid from patients with endometriosis. Fertil. Steril..

[98] Mishra A., Reddy I.J., Gupta P., Mondal S. (2016). Developmental regulation and modulation of apoptotic genes expression in sheep oocytes and embryos cultured in vitro with L-carnitine. Reprod. Domest. Anim..

[99] Reader K.L., Cox N.R., Stanton J.-A.L., Juengel J.L. (2015). Effects of acetyl-L-carnitine on lamb oocyte blastocyst rate, ultrastructure, and mitochondrial DNA copy number. Theriogenology.

[100] Xu H.-Y., Geng S.-S., Li T.-T., Fu Q., Lu S.-S., Liang X.-W., Lu Y.-Q., Zhang M., Yang X.-G., Lu K.-H. (2019). Maturation of buffalo oocytes in vitro with acetyl-L-carnitine improves cryotolerance due to changes in mitochondrial function and the membrane lipid profile. Reprod. Fertil. Dev..

[101] Zhang Q., Wang S.-M., Yao P.-B., Zhang Y.-J., Chen R.-X., Fu Y., Zhang J.-M. (2015). Effects of L-carnitine on follicular survival and graft function following auto transplantation of cryopreserved-thawed ovarian tissues. Cryobiology.

[102] Zarbakhsh S., Safari R., Sameni H.R., Yousefi B., Safari M., Khanmohammadi N., Hayat P. (2019). Effects of Co-Administration of Bone Marrow Stromal Cells and L-carnitine on The Recovery of Damaged Ovaries by Performing Chemotherapy Model in Rat. Int. J. Fertil. Steril..

[103] Somfai T., Kaneda M., Akagi S., Watanabe S., Haraguchi S., Mizutani E., Dang-Nguyen T.Q., Geshi M., Kikuchi K., Nagai T. (2011). Enhancement of lipid metabolism with L-carnitine during in vitro maturation improves nuclear maturation and cleavage ability of follicular porcine oocytes. Reprod. Fertil. Dev..

[104] Zare Z., Farahani R.M., Salehi M., Piryaei A., Novin M.G., Fathabadi F.F., Mohammadi M., Dehghani-Mohammadabadi M. (2015). Effect of L-carnitine supplementation on maturation and early embryo development of immature mouse oocytes selected by brilliant cresyle blue staining. J. Assist. Reprod. Genet..

[105] Jiang W., Li Y., Zhao Y., Gao Q., Jin Q., Yan C., Xu Y. (2020). L-carnitine supplementation during in vitro culture regulates oxidative stress in embryos from bovine aged oocytes. Theriogenology.

[106] You J., Lee J., Hyun S.-H., Lee E. (2012). L-carnitine treatment during oocyte maturation improves in vitro development of cloned pig embryos by influencing intracellular glutathione synthesis and embryonic gene expression. Theriogenology.

[107] Giudice L.C., Kao L.C. (2004). Endometriosis. Lancet.

[108] Giorgi V.S.I., Da Broi M., de Paz C.C.P., Ferriani R.A., Navarro P.A. (2016). N-Acetyl-Cysteine and L-carnitine Prevent Meiotic Oocyte Damage Induced by Follicular Fluid from Infertile Women with Mild Endometriosis. Reprod. Sci..

[109] Giorgi V.S.I., Ferriani R.A., Navarro P.A. (2021). Follicular Fluid from Infertile Women with Mild Endometriosis Impairs In Vitro Bovine Embryo Development: Potential Role of Oxidative Stress. Rev. Bras. Ginecol. Obstet..

[110] Moawad A., Xu B., Tan S.L., Taketo T. (2014). L-carnitine supplementation during vitrification of mouse germinal vesicle stage-oocytes and their subsequent in vitro maturation improves meiotic spindle configuration and mitochondrial distribution in metaphase II oocytes. Hum. Reprod..

[111] Morimoto N., Hashimoto S., Yamanaka M., Satoh M., Nakaoka Y., Fukui A., Morimoto Y., Shibahara H. (2021). Treatment with Laevo (L)-carnitine reverses the mitochondrial function of human embryos. J. Assist. Reprod. Genet..

[112] Miyamoto K., Sato E.F., Kasahara E., Jikumaru M., Hiramoto K., Tabata H., Katsuragi M., Odo S., Utsumi K., Inoue M. (2010). Effect of oxidative stress during repeated ovulation on the structure and functions of the ovary, oocytes, and their mitochondria. Free Radic. Biol. Med..

[113] Ismail A.M., Hamed A.H., Saso S., Thabet H.H. (2014). Adding L-carnitine to clomiphene resistant PCOS women improves the quality of ovulation and the pregnancy rate. A randomized clinical trial. Eur. J. Obstet. Gynecol. Reprod. Biol..

[114] Samimi M., Jamilian M., Ebrahimi F.A., Rahimi M., Tajbakhsh B., Asemi Z. (2016). Oral carnitine supplementation reduces body weight and insulin resistance in women with polycystic ovary syndrome: A randomized, double-blind, placebo-controlled trial. Clin. Endocrinol..

[115] Salehpour S., Nazari L., Hoseini S., Moghaddam P.B., Gachkar L. (2019). Effects of L-carnitine on Polycystic Ovary Syndrome. JBRA Assist. Reprod..

[116] El Sharkwy I., El-Din M.S. (2019). L-carnitine plus metformin in clomiphene-resistant obese PCOS women, reproductive and metabolic effects: A randomized clinical trial. Gynecol. Endocrinol..

[117] Genazzani A.D., Prati A., Genazzani A.R., Battipaglia C., Simoncini T., Szeliga A., Podfigurna A., Meczekalski B. (2020). Synergistic effects of the integrative administration of acetyl-L-carnitine, L-carnitine, L-arginine and N-acetyl-cysteine on metabolic dynamics and on hepatic insulin extraction in overweight/obese patients with PCOS. Gynecol. Reprod. Endocrinol. Metab..

[118] Tauqir S., Israr M., Rauf B., Malik M.O., Habib S.H., Shah F.A., Usman M., Raza M.A., Shah I., Badshah H. (2021). Acetyl-L-carnitine Ameliorates Metabolic and Endocrine Alterations in Women with PCOS: A Double-Blind Randomized Clinical Trial. Adv. Ther..

[119] Jamilian M., Foroozanfard F., Mirhosseini N., Kavossian E., Aghadavod E., Bahmani F., Ostadmohammadi V., Kia M., Eftekhar T., Ayati E. (2019). Effects of Melatonin Supplementation on Hormonal, Inflammatory, Genetic, and Oxidative Stress Parameters in Women with Polycystic Ovary Syndrome. Front. Endocrinol..

[120] Jamilian M., Foroozanfard F., Kavossian E., Kia M., Aghadavod E., Amirani E., Asemi Z. (2020). Effects of Chromium and Carnitine Co-supplementation on Body Weight and Metabolic Profiles in Overweight and Obese Women with Polycystic Ovary Syndrome: A Randomized, Double-Blind, Placebo-Controlled Trial. Biol. Trace Elem. Res..

[121] Kalhori Z., Mehranjani M.S., Azadbakht M., Shariatzadeh M.A. (2019). L-carnitine improves endocrine function and folliculogenesis by reducing inflammation, oxidative stress and apoptosis in mice following induction of polycystic ovary syndrome. Reprod. Fertil. Dev..

[122] Di Emidio G., Rea F., Placidi M., Rossi G., Cocciolone D., Virmani A., Macchiarelli G., Palmerini M.G., D’alessandro A.M., Artini P.G. (2020). Regulatory Functions of L-Carnitine, Acetyl, and Propionyl L-carnitine in a PCOS Mouse Model: Focus on Antioxidant/Antiglycative Molecular Pathways in the Ovarian Microenvironment. Antioxidants.

[123] Durazzo A., Lucarini M., Nazhand A., Souto S.B., Silva A.M., Severino P., Souto E.B., Santini A. (2020). The Nutraceutical Value of Carnitine and Its Use in Dietary Supplements. Molecules.

[124] Di Emidio G., Santini S.J., D’Alessandro A.M., Vetuschi A., Sferra R., Artini P.G., Carta G., Falone S., Amicarelli F., Tatone C. (2019). SIRT1 participates in the response to methylglyoxal-dependent glycative stress in mouse oocytes and ovary. Biochim. Biophys. Acta Mol. Basis Dis..

